# Genetic variants predicting aerobic capacity response to training are also associated with skeletal muscle oxidative capacity in moderate-to-severe COPD

**DOI:** 10.1152/physiolgenomics.00140.2017

**Published:** 2018-05-25

**Authors:** Alessandra Adami, Brian D. Hobbs, Merry-Lynn N. McDonald, Richard Casaburi, Harry B. Rossiter, for the COPDGene Investigators

**Affiliations:** ^1^Rehabilitation Clinical Trials Center, Division of Pulmonary and Critical Care, Physiology and Medicine, Los Angeles Biomedical Research Institute at Harbor-UCLA Medical Center, Torrance, California; ^2^Department of Kinesiology, University of Rhode Island, Kingston, Rhode Island; ^3^Channing Division of Network Medicine, Brigham and Women’s Hospital, Boston, Massachusetts; ^4^Division of Pulmonary and Critical Care Medicine, Brigham and Women’s Hospital, Boston, Massachusetts; ^5^Division of Pulmonary, Allergy and Critical Care Medicine, University of Alabama at Birmingham, Birmingham, Alabama; ^6^Faculty of Biological Sciences, University of Leeds, Leeds, United Kingdom

**Keywords:** exercise, mitochondria, near-infrared spectroscopy, physical activity, single nucleotide polymorphism

## Abstract

Muscle oxidative capacity is a major determinant of maximum oxygen uptake (V̇O_2max_). V̇O_2max_ predicts survival in humans. Muscle oxidative capacity is low in chronic obstructive pulmonary disease (COPD) and can be assessed from the muscle oxygen consumption recovery rate constant (*k*) by near-infrared spectroscopy. We hypothesized that 11 SNPs, previously associated with the increase in V̇O_2max_ following exercise training, would correlate with *k* in 152 non-Hispanic White and African American smokers with and without COPD. Associations were adjusted for age, weight, FEV_1_% predicted, steps/day, and principal components of genetic ancestry. No SNPs were significantly associated with *k*. rs2792022 within *BTAF1* (β = 0.130, *P* = 0.053) and rs24575771 within *SLC22A3* (β = 0.106, *P* = 0.058) approached nominal significance. Case-control stratification identified three SNPs nominally associated with *k* in moderate-to-severe COPD (rs6481619 within *SVIL* β = 0.152, *P* = 0.013; *BTAF1* β = 0.196, *P* = 0.046; rs7386139 within *DEPTOR* β = 0.159, *P* = 0.047). These data support further study of the genomic contributions to skeletal muscle dysfunction in COPD.

## BACKGROUND/MOTIVATION FOR THE STUDY

Low maximum oxygen uptake (V̇O_2max_) holds stronger mortality risk than other established variables such as smoking, obesity or diabetes. Positive adaptation in V̇O_2max_ following increased physical activity is a key inherited trait promoting survival. Plasticity in skeletal muscle oxidative capacity plays a major role in mediating this response (3). The HERITAGE study of 99 families that completed a 20 wk endurance training program identified ~50% heritability of V̇O_2max_ plasticity (2). Later, Timmons et al. (5) identified in muscle biopsies an mRNA expression-based molecular classifier of 29 genes predicting the training response and which were associated with 11 single nucleotide polymorphisms (SNPs) in the HERITAGE cohort. Approximately half of the genetic variance for V̇O_2max_ gained by exercise training was accounted for by these 11 SNPs.

Chronic obstructive pulmonary disease (COPD) is characterized by dyspnea, chronic inactivity, and negative muscle adaptations: major among these is loss of muscle oxidative capacity. Detrimental peripheral adaptations increase ventilatory demand during activities of daily living and are associated with reduced quality of life, morbidity, and mortality in COPD. While genomic and epigenomic variations in oxidative phosphorylation genes have been studied widely in chronic diseases, genetic association studies to explain low muscle oxidative capacity in humans have not. We therefore determined whether the 11 SNPs associated with muscle plasticity in healthy Caucasians were associated with variability in muscle oxidative capacity in non-Hispanic White (NHW) and African American (AA) smokers with and without COPD. Identifying new genetic loci associated muscle adaptations in COPD could potentially identify patients who may benefit most from pulmonary rehabilitation.

## PHENOTYPE

### 

#### Cohort details.

Participants were NHW (*n* = 84) and AA (*n* = 68) aged 45–80 yr with a >10 pack-year smoking history from the COPDGene population cohort study (4). Exclusion criteria included non-COPD respiratory disorders, known or suspected lung cancer or other cancer, lung volume reduction, pregnancy, radiation therapy to the chest, chest or abdominal surgery (past 3 mo), heart attack (past 3 mo), hospitalization (past month). The 243 participants gave written informed consent to participate in the COPDGene Muscle Health ancillary study at LABioMed/Harbor-UCLA (Appendix 1, Supplemental Fig. S1). (The online version of this article contains supplemental material.) The primary trait of gastrocnemius oxidative capacity was determined from the muscle V̇O_2_ recovery rate constant (*k*) using near-infrared spectroscopy (1). Physical activity (steps/day) was assessed during the following 7 days by triaxial accelerometry (DynaPort MoveMonitor, McRoberts). Complete data from 84 COPD (66 ± 9 yr) and 68 smokers with normal spirometry (61 ± 9 yr) were used for final analyses (Appendix 2, Supplemental Table S1). Additional details are provided in Appendix 3.

#### Type of study.

Candidate SNP study.

#### Details of the SNP(s) studied.

Candidate SNPs were identified from Timmons et al. (5) representing 11 different genetic loci (see Appendix 3, Supplemental Table S1 for complete list). Genotyping for the COPDGene study was performed on the Illumina Human Omni-1 Quad array with imputation using the Haplotype Reference Consortium on the Michigan Imputation Server. Quality control details of the COPDGene genotyping data are given in Appendix 2, Supplemental Table S2. Four of the investigated SNPs were imputed: rs3770991 within NRP2; rs4257918 within CPVL; rs2251375 within H19; rs1546570 within DIS3L (Appendix 3, Supplemental Table S1). SNPs were annotated using wANNOVAR (see Appendix 3).

#### Analysis model.

COPD was defined as forced expiratory volume in 1 s/forced vital capacity (FEV_1_/FVC) <0.7, while controls had FEV_1_/FVC > 0.7. In a secondary analysis, moderate-to-severe COPD cases were defined as FEV_1_/FVC < 0.7 and FEV_1_ < 80% predicted. Student’s *t*-tests between controls and COPD were used to identify differences in *k* (positively dependent upon muscle oxidative capacity) or habitual physical activity from number of steps/day. Linear regression with an additive genetic model was performed to identify association of *k* with each of the 11 SNPs, adjusting for age, weight, FEV_1_% predicted, steps/day, and principal components of genetic ancestry using PLINK2.0. Stratified models in moderate-to-severe COPD cases and controls were also evaluated for association with *k*. NHW and AA individuals were analyzed separately; results were then combined in a fixed-effects meta-analysis in METAL, resulting in a total sample size of 152 in the fully adjusted model. Bonferroni-corrected significance was accepted at *P* < 0.0045, and nominal significance was defined as *P* < 0.05.

## RESULTS

COPD patients had lower muscle *k* than controls (mean ± SD in all COPD vs controls, 1.26 ± 0.37 min^−1^ vs. 1.60 ± 0.44 min^−1^, *P* < 0.0001). Steps/day did not differ between COPD and controls (5,740 ± 4,726 vs. 6,509 ± 3,294; *P* = 0.24) (Appendix 1, Supplemental Fig. S2).

No SNPs were significantly associated with *k* in meta-analysis (Appendix 2, Supplemental Table S3). rs2792022 within *BTAF1* [effect allele frequency (EAF) = 0.819, β = 0.130, *P* = 0.053] and rs24575771 within *SLC22A3* (EAF = 0.352, β = 0.106, *P* = 0.058) tended toward nominal significance. Subsequent analysis, stratified by disease state, identified three SNPs nominally associated with *k* in moderate-to-severe COPD only (rs6481619 within *SVIL*, EAF = 0.746, β = 0.152, *P* = 0.013; *BTAF1*, EAF = 0.798, β = 0.196, *P* = 0.046, I^2^ = 41.6; Het_*P* = 0.191; rs7386139 within *DEPTOR*, EAF = 0.219, β = 0.159, *P* = 0.047; Appendix 2, Supplemental Tables S4 and S5). Forest plots stratified by genetic ancestry and disease state are in Appendix 3, Supplemental Figures S1–S3. Two other SNPs tended toward nominal significance in COPD (*P* < 0.1; Appendix 2, Supplemental Table S5). Results of all models stratified by genetic ancestry are in Appendix 3, Supplemental Tables S2–S4.

## INTERPRETATION

This study of 152 smokers is the first to associate SNPs [from candidates identified by Timmons et al. (5)] with muscle oxidative capacity in humans. Nominal association of *k* in moderate-to-severe COPD patients with a SNP intrinsic to *DEPTOR* is of particular interest as this gene is involved with molecular, metabolic, and contractile properties characteristic of glycolytic muscle. As our models adjusted for pulmonary function (FEV_1_) and physical activity (daily steps), the finding of nominally associated SNPs with *k* in moderate-to-severe COPD subjects only, and not in controls, suggests an interaction of these variants with some as-yet-unidentified aspect of COPD. Validation of these candidates may identify new risk factors for symptoms and/or poor outcomes in COPD and other lung diseases where peripheral deconditioning and low muscle oxidative capacity contributes significantly to disease burden. Given that pulmonary rehabilitation is the most beneficial treatment for severe COPD, these SNPs may provide a means of personalizing COPD therapy. These data strengthen the rationale for a larger study to identify potential contributions of genetic variants to low muscle oxidative capacity in COPD patients.

## GRANTS

National Heart, Lung, and Blood Institute (NHLBI) Grants HL-089856 and HL-089897; National Center for Advancing Translational Sciences UCLA CTSI Grant UL1TR000124; Swiss National Science Foundation P300P3_151705 (A. Adami); Swiss National Science Foundation P300PB_167767 (A. Adami); American Thoracic Society Foundation/Breathe LA Project Grant ATS-2014-03 (H. B. Rossiter); NHLBI Grants K08 HL-136928 (B. D. Hobbs) and R00HL-121087 (M. N. McDonald); Parker B. Francis Research Opportunity Award (B. D. Hobbs); Parker B. Francis Foundation Fellowship (M. N. McDonald).

## DISCLOSURES

No conflicts of interest, financial or otherwise, are declared by the authors.

## AUTHOR CONTRIBUTIONS

A.A., R.C., and H.B.R. conceived and designed research; A.A. performed experiments; A.A., B.D.H., and M.N.M. analyzed data; A.A., B.D.H., and H.B.R. interpreted results of experiments; A.A. prepared figures; A.A. and H.B.R. drafted manuscript; A.A., B.D.H., M.N.M., R.C., and H.B.R. edited and revised manuscript; A.A., B.D.H., M.N.M., R.C., and H.B.R. approved final version of manuscript.

## Supplemental Data

Appendix 1 Appendix 1 Figures S1 and S2 (.pdf 141 KB)

Appendix 2 Appendix 2 Tables S1-S5 (.pdf 132 KB)

Appendix 3 Appendix 3: List of Consortium Members, Materials and Methods, Tables S1-S4, Figures S1-S3, References (.pdf 275 KB)

## References

[1] Adami A, Cao R, Porszasz J, Casaburi R, Rossiter HB Reproducibility of NIRS assessment of muscle oxidative capacity in smokers with and without COPD. Respir Physiol Neurobiol 235: 18–26, 2017. doi:10.1016/j.resp.2016.09.008. 27659351 PMC5136338

[2] Bouchard C, Rankinen T, Chagnon YC, Rice T, Pérusse L, Gagnon J, Borecki I, An P, Leon AS, Skinner JS, Wilmore JH, Province M, Rao DC Genomic scan for maximal oxygen uptake and its response to training in the HERITAGE Family Study. J Appl Physiol (1985) 88: 551–559, 2000. doi:10.1152/jappl.2000.88.2.551. 10658022

[3] Hamilton MT, Booth FW Skeletal muscle adaptation to exercise: a century of progress. J Appl Physiol (1985) 88: 327–331, 2000. doi:10.1152/jappl.2000.88.1.327. 10642397

[4] Regan EA, Hokanson JE, Murphy JR, Make B, Lynch DA, Beaty TH, Curran-Everett D, Silverman EK, Crapo JD Genetic epidemiology of COPD (COPDGene) study design. COPD 7: 32–43, 2010. doi:10.3109/15412550903499522. 20214461 PMC2924193

[5] Timmons JA, Knudsen S, Rankinen T, Koch LG, Sarzynski M, Jensen T, Keller P, Scheele C, Vollaard NBJ, Nielsen S, Akerström T, MacDougald OA, Jansson E, Greenhaff PL, Tarnopolsky MA, van Loon LJC, Pedersen BK, Sundberg CJ, Wahlestedt C, Britton SL, Bouchard C Using molecular classification to predict gains in maximal aerobic capacity following endurance exercise training in humans. J Appl Physiol (1985) 108: 1487–1496, 2010. doi:10.1152/japplphysiol.01295.2009. 20133430 PMC2886694

