# Supplementary material for: Genetic variants predicting aerobic capacity response to training are also associated with skeletal muscle oxidative capacity in moderate-to-severe COPD
Source: Physiol Genomics. 2018 May 25;50(9):688–90. doi: 10.1152/physiolgenomics.00140.2017 (PMC6172613; doi:10.1152/physiolgenomics.00140.2017)
Supplement: Appendix 1 — Figures S1 and S2 (.pdf 141 KB) [file Appendix1-figures.pdf]

Appendix 1

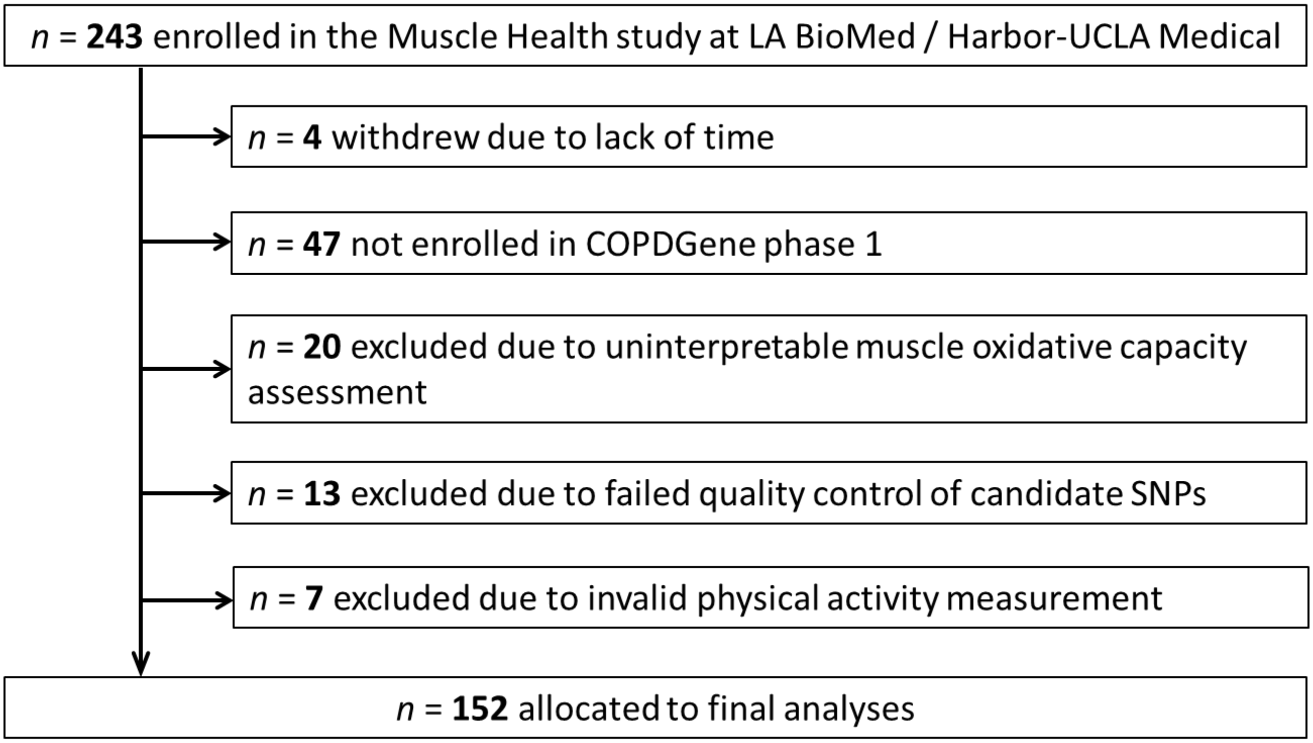

Figure S1. Participants enrolled and reported from the Muscle Health ancillary study of COPDGene (ClinicalsTrials.gov Identifier NCT00608764).

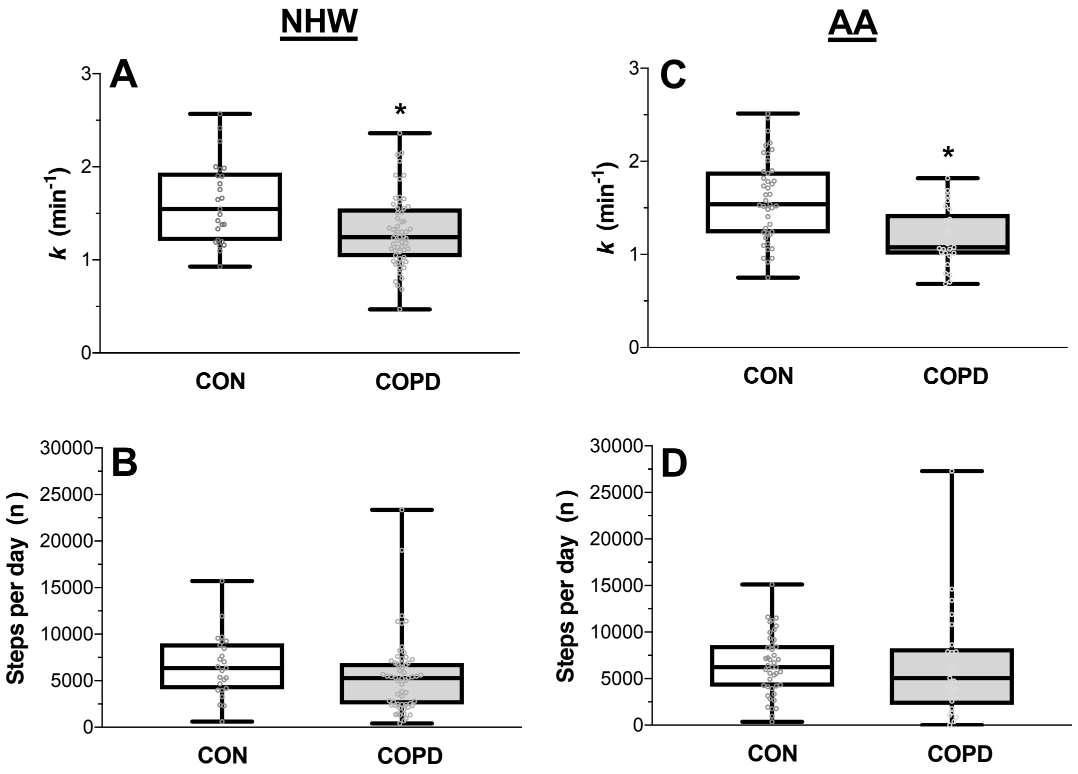

Figure S2. Comparison of muscle *k* (inferred from the muscle oxygen consumption recovery kinetics) (A, C) and number of steps per day (B, D) in non-Hispanic White (NHW) and African American (AA) smokers with normal spirometry (CON) and smokers with COPD. *$p<0.01$.
