# Supplementary material for: Genetic variants predicting aerobic capacity response to training are also associated with skeletal muscle oxidative capacity in moderate-to-severe COPD
Source: Physiol Genomics. 2018 May 25;50(9):688–90. doi: 10.1152/physiolgenomics.00140.2017 (PMC6172613; doi:10.1152/physiolgenomics.00140.2017)
Supplement: Appendix 2 — Tables S1-S5 (.pdf 132 KB) [file Appendix2-tables.pdf]

Appendix 2 - Tables

Table S1. Participant characteristics.

	NHW (n= 84)			AA (n = 68)		
	CON	COPD	p-value	CON	COPD	p-value
n	25	59	-	43	25	-
Current smoker (n (%))	3 (12)	14 (24)	-	34 (79)	14 (56)	-
Age (yrs)	67 (10)	68 (10)	NS	57 (5) $*	62 (7) *	NS
Weight (kg)	79 (20)	78 (17)	NS	87 (21)	81 (16)	NS
Height (cm)	167 (8)	168 (11)	NS	171 (10)	173 (8)	NS
BMI (kg/m^2)	28 (7)	28 (6)	NS	30 (7)	27 (5)	NS
Sex (M/F)	9/16	25/34	-	24/19	20/5	-
FVC (L)	3.3 (0.8)	3.1 (1.0)	NS	3.3 (0.8)	3.0 (0.8)	NS
FEV_1 (L)	2.5 (0.6)	1.7 (0.8) #	NS	2.6 (0.6)	1.8 (0.7) $	NS
FEV_1 %pred (%)	98.9 (19.8)	65.1 (26.7) #	NS	97.9 (13.0)	64.6 (22.7) $	NS
k (min^{-1})	1.62 (0.09)	1.30 (0.05) #	0.007	1.59 (0.07)	1.18 (0.06) $	0.0001
Step/day (n)	6,589 (665)	5,446 (538)	NS	6.463 (505)	6,434 (1,188)	NS

Data are mean (SD). NHW = non-Hispanic White; AA = African American; CON= smoker controls with normal spirometry; COPD = chronic obstructive pulmonary disease; BMI = body mass index; M = male; F = female; FVC = forced vital capacity; FEV_1 = forced expiratory volume in 1 second; k = muscle oxygen consumption recovery rate constant, proportional to muscle oxidative capacity. p≤0.05 vs: $ NHW CON; * NHW COPD; # AA CON

Table S2. Quality control of COPDGene genotyping.

Study genotyping / QC							SNP QC				
**Cohort Name**	**Array used and version**	**Genotype calling software**	**Related individuals (yes/no)?**	**Familial adjustment method**	**Population stratification assessment and adjustment**	**Analysis software version**	**MAF**	**Call Rate**	**HWE p-value**	**# SNPs analysed post-QC**	**Other filtering**
COPDGene	Illumina Human Omni 1-Quad (Restricted to candidate SNPs)	*Illumina (GenCall)*	*no*	*NA*	*Exclusion based on PCA. Models adjusted for PCs for genetic ancetry*	*PLINK v1.9*	*NA*	*>98%*	*>10-8*	*11*	*No*

Imputation information						Sample QC	
**Number of SNPs used for imputation**	**Imputation software**	**Imputation backbone**	**Haplotypes used for backbone**	**NCBI build**	**Chr X imputed yes/no**	**Call Rate**	**Other QC exclusions**
Illumina Human Omni 1-Quad	*Michigan Imputation Server*	*Haplotype Reference Consortium*	*All*	*37*	*No*	*>98.5%*	*IBS, pop strat, heterozygosity (Cho et al. Lancet Resp Med 3(4):264-6, 2015)*

Table S3. Associations between candidate SNPs and muscle oxidative capacity inferred from *gastrocnemius* oxygen consumption recovery rate constant (*k*) in the complete cohort (n=152). Associations were corrected for age, weight, FEV_1 %predicted, number of steps per day, and the principal components of genetic ancestry.

rsID	Locus name	Chr	Position	EA/ALT	Trait	EAF	β (SE)	*P*	N	I^2	Het_*P*
rs11574	*ID3* (nonsynonymous)	1	23,885,498	C/T	Gastroc *k*	0.829	0.024 (0.064)	0.707	152	74.4	0.0481
rs10497520	*TTN* (nonsynonymous)	2	179,644,855	T/C	Gastroc *k*	0.309	0.109 (0.062)	0.079	152	0	0.4725
rs3770991	*NRP2* (intronic)	2	206,655,739	A/G	Gastroc *k*	0.152	0.062 (0.076)	0.413	152	43.5	0.1833
rs2457571	*SLC22A3* (intronic)	6	160,834,828	T/C	Gastroc *k*	0.352	0.106 (0.056)	0.058	152	0	0.6574
rs4257918	*CPVL* (intronic)	7	29,053,849	A/G	Gastroc *k*	0.152	0.011 (0.07)	0.875	152	0	0.8882
rs7386139	*DEPTOR* (intronic)	8	121,027,419	G/A	Gastroc *k*	0.201	0.036 (0.066)	0.581	152	0	0.7869
rs6481619	*SVIL* (intronic)	10	29,982,954	A/C	Gastroc *k*	0.763	0.059 (0.059)	0.314	152	46.5	0.1717
rs2792022	*BTAF1* (intronic)	10	93,740,429	T/C	Gastroc *k*	0.819	0.13 (0.067)	0.053	152	0	0.9114
rs2251375	*H19* (ncRNA_intronic)	11	2,019,496	C/A	Gastroc *k*	0.520	0.057 (0.052)	0.278	152	0	0.8010
rs7324557	*TNFRSF19;MIPEP* (intergenic)	13	24,296,862	A/G	Gastroc *k*	0.296	0.014 (0.052)	0.795	152	0	0.4145
rs1546570	*DIS3L* (intronic)	15	66,595,775	A/C	Gastroc *k*	0.187	0.111 (0.06)	0.065	152	0	0.5755

Abbreviations: rsID - dbSNP rsID; locus name, variant annotation; Chr - chromosome; Pos - Build 37 position; EA/ALT - Effect allele and alternative allele; Trait, Gastroc *k* - Gastrocnemius muscle oxygen consumption recovery rate constant (*k*); EAF - Effect allele frequency; β (SE)/Z-score - effect estimate and standard error for a quantitative trait and Z-score for example for a binary trait; *P* - *P*-value for association; N - number of samples analyzed; I^2 - Heterogeneity; Het_*P* - *P*-value for heterogeneity between AA and NHW ancestry.

Table S4. Associations between candidate SNPs and muscle oxidative capacity inferred from *gastrocnemius* oxygen consumption recovery rate constant (*k*) in smokers without COPD (n=60). Associations were corrected for age, weight, FEV_1 %predicted, number of steps per day, and the principal components of genetic ancestry.

rsID	Locus name	Chr	Position	EA/ALT	Trait	EAF	β (SE)	*P*	N	I^2	Het_*P*
rs11574	*ID3* (nonsynonymous)	1	23,885,498	C/T	Gastroc *k*	0.891	0.075 (0.148)	0.613	60	27.5	0.2402
rs10497520	*TTN* (nonsynonymous)	2	179,644,855	T/C	Gastroc *k*	0.458	0.044 (0.089)	0.618	60	0	0.3343
rs3770991	*NRP2* (intronic)	2	206,655,739	A/G	Gastroc *k*	0.784	0.01 (0.11)	0.930	60	0	0.5572
rs2457571	*SLC22A3* (intronic)	6	160,834,828	T/C	Gastroc *k*	0.250	0.191 (0.104)	0.068	60	0	0.5038
rs4257918	*CPVL* (intronic)	7	29,053,849	A/G	Gastroc *k*	0.134	0.055 (0.113)	0.625	60	0	0.5910
rs7386139	*DEPTOR* (intronic)	8	121,027,419	G/A	Gastroc *k*	0.817	0.048 (0.104)	0.643	60	20.1	0.2634
rs6481619	*SVIL* (intronic)	10	29,982,954	A/C	Gastroc *k*	0.767	0.089 (0.097)	0.360	60	0	0.4534
rs2792022	*BTAF1* (intronic)	10	93,740,429	T/C	Gastroc *k*	0.883	0.128 (0.123)	0.299	60	0	0.9642
rs2251375	*H19* (ncRNA_intronic)	11	2,019,496	C/A	Gastroc *k*	0.435	0.096 (0.082)	0.241	60	26.6	0.2433
rs7324557	*TNFRSF19;MIPEP* (intergenic)	13	24,296,862	A/G	Gastroc *k*	0.258	0.05 (0.089)	0.579	60	0	0.7677
rs1546570	*DIS3L* (intronic)	15	66,595,775	A/C	Gastroc *k*	0.225	0.131 (0.091)	0.149	60	0	0.7485

Abbreviations: rsID - dbSNP rsID; locus name, variant annotation; Chr - chromosome; Pos - Build 37 position; EA/ALT - Effect allele and alternative allele; Trait, Gastroc *k* - Gastrocnemius muscle oxygen consumption recovery rate constant (*k*); EAF - Effect allele frequency; β (SE)/Z-score - effect estimate and standard error for a quantitative trait and Z-score for example for a binary trait; *P* - *P*-value for association; N - number of samples analysed; I^2 - Heterogeneity; Het_*P* - *P*-value for heterogeneity between AA and NHW ancestry.

Table S5. Associations between candidate SNPs and muscle oxidative capacity inferred from *gastrocnemius* oxygen consumption recovery rate constant (*k*) in smokers with moderate to severe COPD (n=57). Associations were corrected for age, weight, FEV_1 %predicted, number of steps per day, and the principal components of genetic ancestry.

rsID	Locus name	Chr	Position	EA/ALT	Trait	EAF	β (SE)	*P*	N	I^2	Het_*P*
rs11574	*ID3* (nonsynonymous)	1	23,885,498	C/T	Gastroc *k*	0.789	0.09 (0.072)	0.209	57	83.4	0.0141
rs10497520	*TTN* (nonsynonymous)	2	179,644,855	T/C	Gastroc *k*	0.211	0.119 (0.071)	0.093	57	0	0.9780
rs3770991	*NRP2* (intronic)	2	206,655,739	A/G	Gastroc *k*	0.099	0.051 (0.087)	0.560	57	9.3	0.2938
rs2457571	*SLC22A3* (intronic)	6	160,834,828	T/C	Gastroc *k*	0.548	0.020 (0.087)	0.820	42	0	1.0000
rs4257918	*CPVL* (intronic)	7	29,053,849	A/G	Gastroc *k*	0.159	0.128 (0.076)	0.094	57	0	0.4917
rs7386139	*DEPTOR* (intronic)	8	121,027,419	G/A	Gastroc *k*	0.219	0.159 (0.08)	0.047	57	0	0.4303
rs6481619	*SVIL* (intronic)	10	29,982,954	A/C	Gastroc *k*	0.746	0.152 (0.061)	0.013	57	0	0.5385
rs2792022	*BTAF1* (intronic)	10	93,740,429	T/C	Gastroc *k*	0.789	0.196 (0.098)	0.046	57	41.6	0.1906
rs2251375	*H19* (ncRNA_intronic)	11	2,019,496	C/A	Gastroc *k*	0.415	0.007 (0.057)	0.900	57	88	0.0039
rs7324557	*TNFRSF19;MIPEP* (intergenic)	13	24,296,862	A/G	Gastroc *k*	0.710	0.066 (0.066)	0.312	57	0	0.4190
rs1546570	*DIS3L* (intronic)	15	66,595,775	A/C	Gastroc *k*	0.149	0.075 (0.112)	0.502	57	0	0.6657

Abbreviations: rsID - dbSNP rsID; locus name, variant annotation; Chr - chromosome; Pos - Build 37 position; EA/ALT - Effect allele and alternative allele; Trait, Gastroc *k* - Gastrocnemius muscle oxygen consumption recovery rate constant (*k*); EAF - Effect allele frequency; β (SE)/Z-score - effect estimate and standard error for a quantitative trait and Z-score for example for a binary trait; *P* - *P*-value for association; N - number of samples analysed; I^2 - Heterogeneity; Het_*P* - *P*-value for heterogeneity between AA and NHW ancestry.
