# Supplementary material for: Genetic variants predicting aerobic capacity response to training are also associated with skeletal muscle oxidative capacity in moderate-to-severe COPD
Source: Physiol Genomics. 2018 May 25;50(9):688–90. doi: 10.1152/physiolgenomics.00140.2017 (PMC6172613; doi:10.1152/physiolgenomics.00140.2017)
Supplement: Appendix 3 — List of Consortium Members, Materials and Methods, Tables S1-S4, Figures S1-S3, References (.pdf 275 KB) [file Appendix3-suppl.pdf]

Appendix 3: Supplemental Materials

LIST OF CONSORTIUM MEMBERS (in alphabetical order)

Adami Alessandra, Rehabilitation Clinical Trials Center, Division of Pulmonary and Critical Care Physiology and Medicine, Los Angeles Biomedical Research Institute at Harbor-UCLA Medical Center, Torrance, CA, USA; and Department of Kinesiology, University of Rhode Island, Kingston, RI, USA. (aadami@uri.edu)

Richard Casaburi, Rehabilitation Clinical Trials Center, Division of Pulmonary Critical Care Physiology and Medicine, Los Angeles Biomedical Research Institute at Harbor-UCLA Medical Center, Torrance, CA, USA. (casaburi@ucla.edu)

Michael H. Cho, Channing Division of Network Medicine, Brigham and Women's Hospital, Boston, MA, USA; and Division of Pulmonary and Critical Care Medicine, Brigham and Women's Hospital, Boston, MA, USA. (remhc@channing.harvard.edu)

Brian D. Hobbs, Channing Division of Network Medicine, Brigham and Women's Hospital, Boston, MA, USA; and Division of Pulmonary and Critical Care Medicine, Brigham and Women's Hospital, Boston, MA, USA. (rebdh@channing.harvard.edu)

Merry-Lynn McDonald, Division of Pulmonary, Allergy and Critical Care Medicine, University of Alabama at Birmingham, Birmingham, AL, USA. (mldonnelly@uabmc.edu)

Janos Porszasz, Rehabilitation Clinical Trials Center, Division of Pulmonary and Critical Care Physiology and Medicine, Los Angeles Biomedical Research Institute at Harbor-UCLA Medical Center, Torrance, CA, USA. (porszasz@ucla.edu)

Harry B. Rossiter, Rehabilitation Clinical Trials Center, Division of Pulmonary and Critical Care Physiology and Medicine, Los Angeles Biomedical Research Institute at Harbor-UCLA Medical Center, Torrance, CA, USA; and Faculty of Biological Sciences University of Leeds, Leeds, UK. (hrossiter@ucla.edu)

Edwin K. Silverman, Channing Division of Network Medicine, Brigham and Women's Hospital, Boston, MA, USA; and Division of Pulmonary and Critical Care Medicine, Brigham and Women's Hospital, Boston, MA, USA. (reeks@channing.harvard.edu)

William Stringer, Rehabilitation Clinical Trials Center, Division of Pulmonary and Critical Care Physiology and Medicine, Los Angeles Biomedical Research Institute at Harbor-UCLA Medical Center, Torrance, CA, USA. (stringer@ucla.edu)

For the COPDGene Investigators

MATERIALS AND METHODS

Each participant visited the laboratory once for approximately 3 hours. The visit included medical and smoking history questionnaires, spirometry, blood sample for genetic sequencing, and muscle oxidative capacity test using near-infrared spectroscopy. At the completion of the visit, each participant was provided with a triaxial accelerometer (Dynaport, McRoberts, NL) to wear in the small of the back and held in place by an elastic belt to determine daily step counts.

Spirometry: Approximately 15 minutes before spirometric testing, participants inhaled two puffs of metered dose albuterol sulfate (ProAir HFA, Teva Respiratory, Horsham, PA, USA). Spirometry was performed in accordance with the American Thoracic Society guidelines (6) using a dual beam Doppler ultrasound-based spirometer (EasyOne Pro, Ndd Medical, Zürich, Switzerland) (9). Forced expiratory volume in 1 second (FEV_1) and forced vital capacity (FVC) were measured from the greatest FEV_1 and FVC over up to eight maximum expiratory maneuvers, where the greatest two measurements were within 150 mL.

Genetic analyses: Genotyping for the COPDGene study was performed on the Illumina Human Omni-1 Quad array with imputation using the Haplotype Reference Consortium on the Michigan Imputation Server. Quality control details of the COPDGene genotyping data is given in Appendix 2, Table 2. SNPs were annotated using wANNOVAR (3, 12, 15). Linear regression with an additive genetic model was performed to identify the association of k with each of the 11 SNPs, adjusting for age, weight, pulmonary function (FEV_1% predicted), physical activity (steps/day) and principal components of genetic ancestry using PLINK2.0 (www.cog-genomics.org/plink/2.0/; 2). Additional models were run without an adjustment for steps/day.

NHW and AA individuals were analyzed separately and results were combined in a fixed-effects meta-analysis in METAL (13) resulting in a total sample size of 152 in the fully adjusted model.

Muscle oxidative capacity: We applied a protocol recently validated on smokers with and without COPD (1) on the medial *gastrocnemius* muscle. A continuous-wave, spatially-resolved spectroscopy (SRS) NIRS device (PortaMon, Artinis, The Netherlands) was used to measure the relative concentrations of oxygenated and deoxygenated hemoglobin (HbO_2 and HHb), and the tissue saturation index was determined using the SRS approach (TSI, %) (4). With the participant lying supine, the NIRS probe was placed longitudinally on the belly of the medial *gastrocnemius*. A rapid-inflation pressure-cuff (SC12D, Hokanson, USA) was placed on the proximal thigh of the same limb and attached to an electronically-controlled rapid cuff-inflator (E20, Hokanson, USA). All participants completed an initial familiarization consisting of a short series of sub-maximal muscle contractions and familiarization with the sensations of rapid-cuff inflation around the thigh from low to high pressures (~50-250 mm Hg). Familiarization was also used to establish arterial occlusion pressure from a tolerated cuff-pressure ~250 mm Hg accompanied by progressive muscle desaturation with constant muscle total hemoglobin (THb = HbO_2 + HHb). The participant was asked to relax and refrain from moving the leg except when instructed. The assessment began with 2-3 minutes of resting baseline measurement of *gastrocnemius* TSI and fingertip pulse oximetry (Rad-5 Pulse Oximeter MasimoSET®, Masimo Co., Irvine, CA). Following this, a physiological normalization was performed with complete arterial occlusion of the thigh until constant (low) TSI was achieved or 5 minutes, whichever was the sooner. This value was used to normalize NIRS signals to the maximum and minimum physiologic range. After ~5 minutes resting to re-establish baseline hemodynamics, the participant performed two muscle oxidative capacity assessments, each consisting of ~10-15 s sub-maximal rhythmic muscle contractions followed by a series of intermittent arterial occlusions (5 occlusions for 5 s, and 10 for 10 s, each separated by 5-20 s recovery). The two muscle oxidative capacity assessments were separated by ~1-2 minutes of resting recovery. Afterwards the adipose tissue thickness was estimated at the NIRS site using a skinfold caliper (Lange Skinfold Caliper, Beta Technology Inc., Santa Cruz, CA). From the NIRS data relative muscle oxygen consumption (in units of % change in TSI per second) was determined during each post-contraction arterial occlusion and was used to construct muscle oxygen consumption recovery kinetics. The exponential recovery rate constant, k, was characterized using non-linear least squares fitting, as preciously described (Origin 8.6, OriginLab, MA, USA; 1). Muscle oxygen consumption recovery k is linearly associated with muscle oxidative capacity (10, 14).

Daily step counts by triaxial accelerometry: Physical activity was assessed objectively using a a portable triaxial accelerometer (DynaPort ADL-monitor, McRoberts BV, NL) that has been validated in healthy, elderly and COPD patients (5, 7, 8, 11). At the end of the clinic visit, participants received instructions on wearing the monitoring device around their waist for 7 consecutive days. Participants were instructed to wear the DynaPort accelerometer for 24 hours per day, and only to remove it for bathing. Valid measurements were determined by at least 94% wearing compliance within a total of at least five 24-hour periods. After completion of the 7th day of wearing the device was delivered back to laboratory for data download and analysis using the manufacturer's algorithms (MoveMonitor version 2.8).

Table S1. Genotyped and imputed SNPs with accuracy of imputation

rsID	Locus name (substitution class)	Chr	Position	Genotyped or Imputed	AA Rsq	NHW Rsq
rs11574	*ID3* (nonsynonymous)	1	23,885,498	genotyped		
rs10497520	*TTN* (nonsynonymous)	2	179,644,855	genotyped		
rs3770991	*NRP2* (intronic)	2	206,655,739	imputed	0.92124	0.99049
rs2457571	*SLC22A3* (intronic)	6	160,834,828	genotyped		
rs4257918	*CPVL* (intronic)	7	29,053,849	imputed	0.98837	0.99902
rs7386139	*DEPTOR* (intronic)	8	121,027,419	genotyped		
rs6481619	*SVIL* (intronic)	10	29,982,954	genotyped		
rs2792022	*BTAF1* (intronic)	10	93,740,429	genotyped		
rs2251375	*H19* (ncRNA_intronic)	11	2,019,496	imputed	0.97513	0.99245
rs7324557	*TNFRSF19;MIPEP* (intergenic)	13	24,296,862	genotyped		
rs1546570	*DIS3L* (intronic)	15	66,595,775	imputed	0.99840	0.99986

Abbreviations: rsID - dbSNP rsID; locus name - variant annotation; Chr - chromosome; Position - Build 37 position; AA Rsq – imputation quality score in African American COPDGene participants, NHW Rsq – imputation quality score in Non-Hispanic White COPDGene participants

Table S2. Associations stratified by ancestry between candidate SNPs and muscle oxidative capacity inferred from *gastrocnemius* oxygen consumption recovery rate constant (*k*) in the complete cohort (n=152). Associations were corrected for age, weight, FEV_1 %predicted, number of steps per day.

						African American				Non-Hispanic White			
**rsID**	**Locus name**	**Chr**	**Position**	**EA/ALT**	**Trait**	**EAF**	**β (SE)**	***P***	**N**	**EAF**	**β (SE)**	***P***	**N**
rs11574	*ID3* (nonsynonymous)	1	23,885,498	C/T	Gastroc *k*	0.939	0.322 (0.164)	0.054	66	0.744	-0.03 (0.07)	0.671	86
rs10497520	*TTN* (nonsynonymous)	2	179,644,855	T/C	Gastroc *k*	0.576	0.141 (0.076)	0.069	66	0.105	0.047 (0.106)	0.655	86
rs3770991	*NRP2* (intronic)	2	206,655,739	A/G	Gastroc *k*	0.239	-0.018 (0.097)	0.857	66	0.086	0.191 (0.123)	0.124	86
rs2457571	*SLC22A3* (intronic)	6	160,834,828	T/C	Gastroc *k*	0.106	0.057 (0.124)	0.647	66	0.541	0.118 (0.063)	0.062	86
rs4257918	*CPVL* (intronic)	7	29,053,849	A/G	Gastroc *k*	0.192	0.003 (0.093)	0.979	66	0.122	0.022 (0.107)	0.835	86
rs7386139	*DEPTOR* (intronic)	8	121,027,419	G/A	Gastroc *k*	0.152	0.012 (0.112)	0.917	66	0.238	0.049 (0.081)	0.547	86
rs6481619	*SVIL* (intronic)	10	29,982,954	A/C	Gastroc *k*	0.795	0.151 (0.089)	0.096	66	0.738	-0.011 (0.079)	0.884	86
rs2792022	*BTAF1* (intronic)	10	93,740,429	T/C	Gastroc *k*	0.947	0.112 (0.178)	0.533	66	0.721	0.133 (0.073)	0.070	86
rs2251375	*H19* (ncRNA_intronic)	11	2,019,496	C/A	Gastroc *k*	0.386	0.041 (0.08)	0.609	66	0.622	0.068 (0.069)	0.326	86
rs7324557	*TNFRSF19;MIPEP* (intergenic)	13	24,296,862	A/G	Gastroc *k*	0.257	0.067 (0.084)	0.426	66	0.326	-0.02 (0.066)	0.763	86
rs1546570	*DIS3L* (intronic)	15	66,595,775	A/C	Gastroc *k*	0.136	0.065 (0.102)	0.525	66	0.227	0.136 (0.075)	0.072	86

Abbreviations: rsID - dbSNP rsID; locus name, variant annotation; Chr - chromosome; Pos - Build 37 position; EA/ALT - Effect allele and alternative allele; Trait, Gastroc *k* - Gastrocnemius muscle oxygen consumption recovery rate constant (*k*); EAF - Effect allele frequency; β (SE)/Z-score - effect estimate and standard error for a quantitative trait and Z-score for example for a binary trait; *P* - *P*-value for association; N - number of samples analyzed

Table S3. Associations stratified by ancestry between candidate SNPs and muscle oxidative capacity inferred from *gastrocnemius* oxygen consumption recovery rate constant (*k*) in smokers without COPD (n=60). Associations were corrected for age, weight, FEV_1 %predicted, number of steps per day.

						African American				Non-Hispanic White			
**rsID**	**Locus name**	**Chr**	**Position**	**EA/ALT**	**Trait**	**EAF**	**β (SE)**	***P***	**N**	**EAF**	**β (SE)**	***P***	**N**
rs11574	*ID3* (nonsynonymous)	1	23,885,498	C/T	Gastroc *k*	0.975	0.409 (0.321)	0.210	40	0.725	-0.016 (0.167)	0.925	20
rs10497520	*TTN* (nonsynonymous)	2	179,644,855	T/C	Gastroc *k*	0.600	0.086 (0.099)	0.390	40	0.175	-0.134 (0.205)	0.522	20
rs3770991	*NRP2* (intronic)	2	206,655,739	A/G	Gastroc *k*	0.746	0.045 (0.125)	0.721	40	0.861	-0.108 (0.229)	0.642	20
rs2457571	*SLC22A3* (intronic)	6	160,834,828	T/C	Gastroc *k*	0.100	0.095 (0.178)	0.597	40	0.550	0.241 (0.129)	0.078	20
rs4257918	*CPVL* (intronic)	7	29,053,849	A/G	Gastroc *k*	0.152	0.087 (0.128)	0.500	40	0.100	-0.062 (0.246)	0.804	20
rs7386139	*DEPTOR* (intronic)	8	121,027,419	G/A	Gastroc *k*	0.825	0.141 (0.133)	0.296	40	0.800	-0.098 (0.168)	0.565	20
rs6481619	*SVIL* (intronic)	10	29,982,954	A/C	Gastroc *k*	0.788	0.141 (0.119)	0.245	40	0.725	-0.013 (0.168)	0.937	20
rs2792022	*BTAF1* (intronic)	10	93,740,429	T/C	Gastroc *k*	0.950	0.119 (0.237)	0.619	40	0.750	0.131 (0.144)	0.374	20
rs2251375	*H19* (ncRNA_intronic)	11	2,019,496	C/A	Gastroc *k*	0.366	0.148 (0.093)	0.121	40	0.575	-0.08 (0.172)	0.647	20
rs7324557	*TNFRSF19;MIPEP* (intergenic)	13	24,296,862	A/G	Gastroc *k*	0.262	0.066 (0.106)	0.534	40	0.250	0.008 (0.167)	0.962	20
rs1546570	*DIS3L* (intronic)	15	66,595,775	A/C	Gastroc *k*	0.162	0.104 (0.125)	0.412	40	0.350	0.162 (0.133)	0.237	20

Abbreviations: rsID - dbSNP rsID; locus name, variant annotation; Chr - chromosome; Pos - Build 37 position; EA/ALT - Effect allele and alternative allele; Trait, Gastroc *k* - Gastrocnemius muscle oxygen consumption recovery rate constant (*k*); EAF - Effect allele frequency; β (SE)/Z-score - effect estimate and standard error for a quantitative trait and Z-score for example for a binary trait; *P* - *P*-value for association; N - number of samples analyzed

Table S4. Associations stratified by ancestry between candidate SNPs and muscle oxidative capacity inferred from *gastrocnemius* oxygen consumption recovery rate constant (*k*) in smokers with moderate to severe COPD (n=57). Associations were corrected for age, weight, FEV_1 %predicted, number of steps per day.

						African American				Non-Hispanic White			
**rsID**	**Locus name**	**Chr**	**Position**	**EA/ALT**	**Trait**	**EAF**	**β (SE)**	***P***	**N**	**EAF**	**β (SE)**	***P***	**N**
rs11574	*ID3* (nonsynonymous)	1	23,885,498	C/T	Gastroc *k*	0.867	0.273 (0.103)	0.020	15	0.762	-0.079 (0.099)	0.432	42
rs10497520	*TTN* (nonsynonymous)	2	179,644,855	T/C	Gastroc *k*	0.500	0.118 (0.083)	0.182	15	0.107	0.122 (0.134)	0.367	42
rs3770991	*NRP2* (intronic)	2	206,655,739	A/G	Gastroc *k*	0.275	0.015 (0.093)	0.873	15	0.036	0.285 (0.24)	0.241	42
rs2457571	*SLC22A3* (intronic)	6	160,834,828	T/C	Gastroc *k*	--	--	--	--	0.548	0.02 (0.087)	0.820	42
rs4257918	*CPVL* (intronic)	7	29,053,849	A/G	Gastroc *k*	0.272	0.095 (0.09)	0.311	15	0.119	0.211 (0.143)	0.148	42
rs7386139	*DEPTOR* (intronic)	8	121,027,419	G/A	Gastroc *k*	0.133	0.221 (0.112)	0.070	15	0.250	0.095 (0.114)	0.408	42
rs6481619	*SVIL* (intronic)	10	29,982,954	A/C	Gastroc *k*	0.700	0.13 (0.071)	0.090	15	0.762	0.216 (0.121)	0.081	42
rs2792022	*BTAF1* (intronic)	10	93,740,429	T/C	Gastroc *k*	0.967	0.417 (0.195)	0.052	15	0.726	0.122 (0.114)	0.292	42
rs2251375	*H19* (ncRNA_intronic)	11	2,019,496	C/A	Gastroc *k*	0.545	0.152 (0.076)	0.067	15	0.369	-0.179 (0.086)	0.044	42
rs7324557	*TNFRSF19;MIPEP* (intergenic)	13	24,296,862	A/G	Gastroc *k*	0.767	0.015 (0.092)	0.875	15	0.690	0.121 (0.094)	0.207	42
rs1546570	*DIS3L* (intronic)	15	66,595,775	A/C	Gastroc *k*	0.033	-0.01 (0.227)	0.964	15	0.190	0.102 (0.128)	0.430	42

Abbreviations: rsID - dbSNP rsID; locus name, variant annotation; Chr - chromosome; Pos - Build 37 position; EA/ALT - Effect allele and alternative allele; Trait, Gastroc *k* - Gastrocnemius muscle oxygen consumption recovery rate constant (*k*); EAF - Effect allele frequency; β (SE)/Z-score - effect estimate and standard error for a quantitative trait and Z-score for example for a binary trait; *P* - *P*-value for association; N - number of samples analyzed

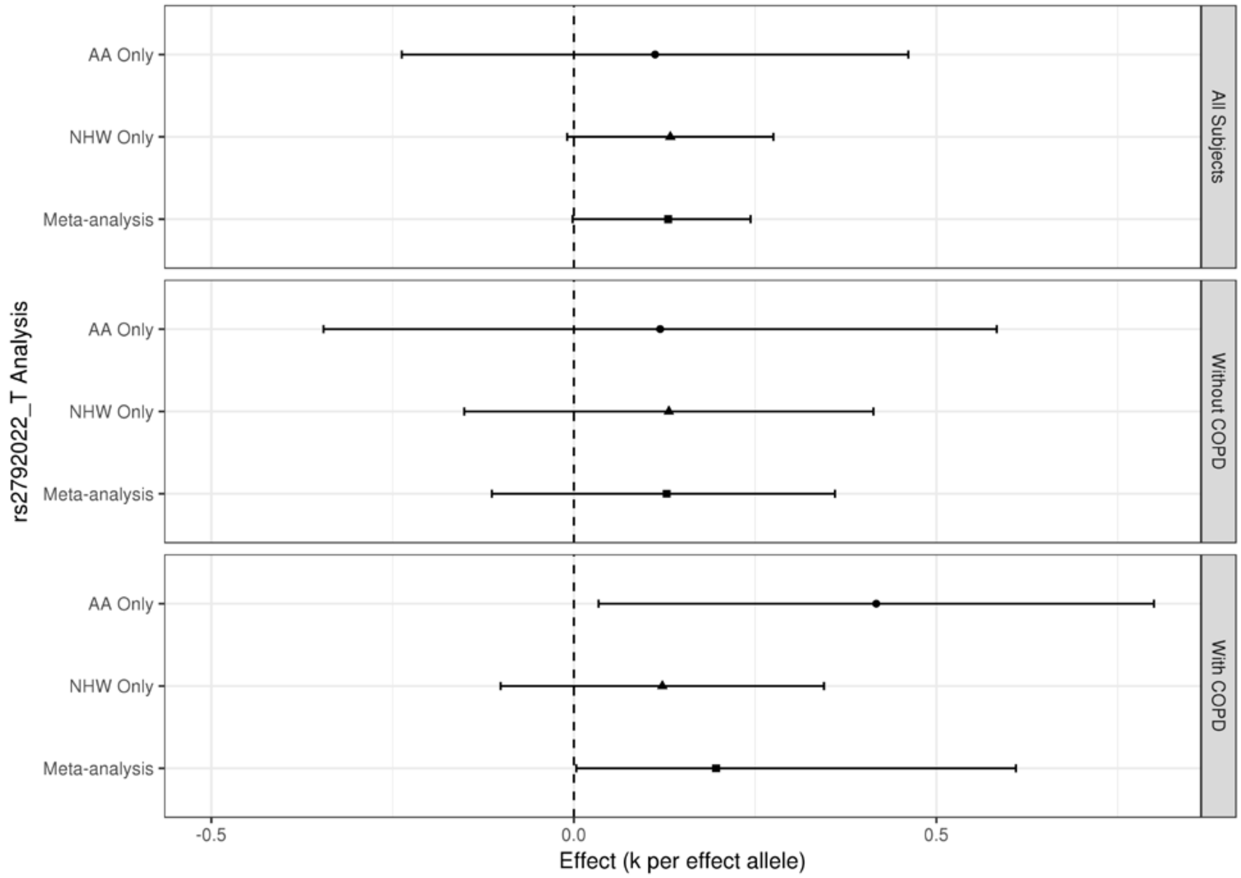

Figure S1. The effect on muscle oxygen consumption recovery rate constant (k) of T/C genetic variation on rs2792022 *BTAF1* among all subjects in study, those without COPD and those with moderate-to-severe COPD stratified by genetic ancestry. AA (circle) = African American. NHW (triangle) = Non-Hispanic White. Meta-analysis between genetic ancestry (square). Subjects with COPD were those with moderate-to-severe disease ($FEV_1/FVC<0.7$ and FEV_1%predicted <80%). Error bars in forest plot show 95% confidence intervals.

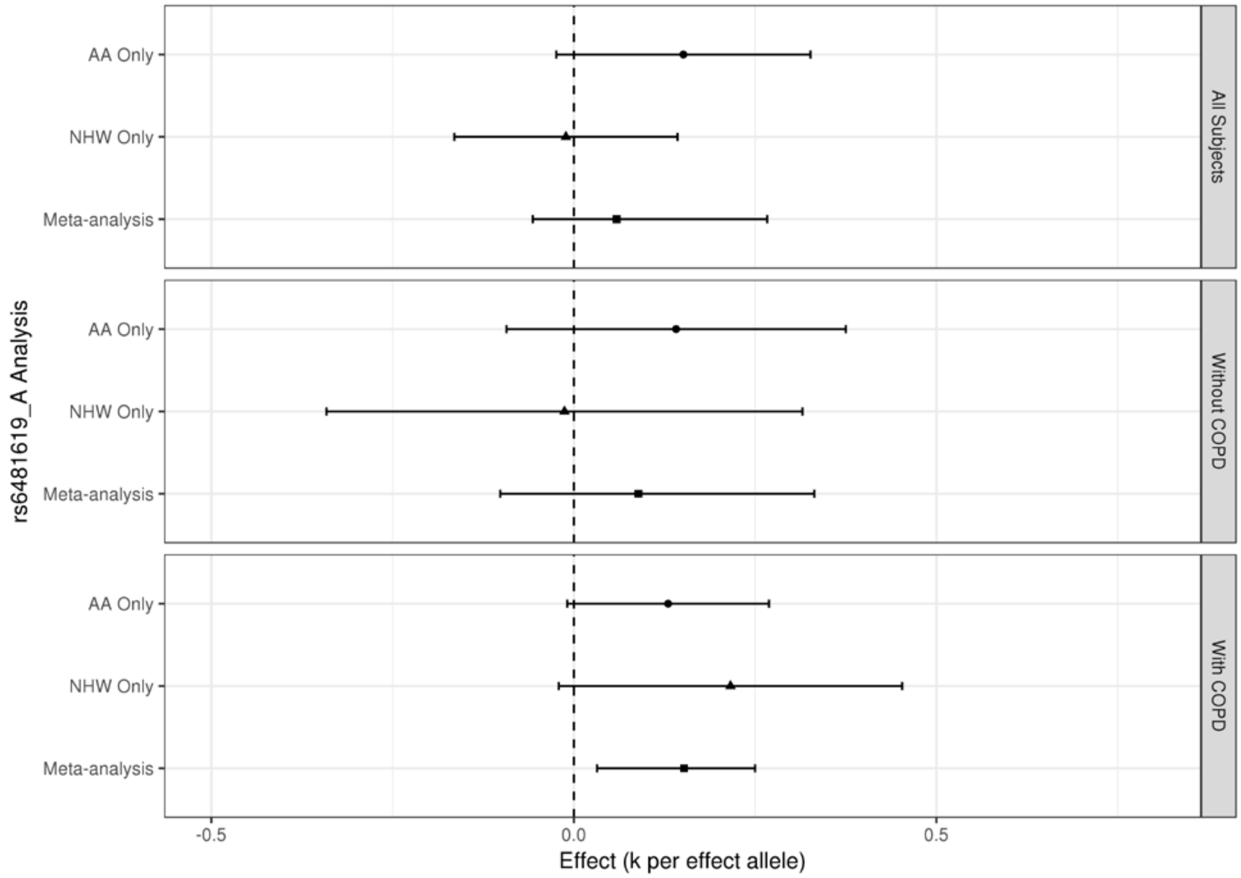

Figure S2. The effect on muscle oxygen consumption recovery rate constant (k) of G/A genetic variation on rs6481619 *SVIL* among all subjects in study, those without COPD and those with moderate-to-severe COPD stratified by genetic ancestry. AA (circle) = African American. NHW (triangle) = Non-Hispanic White. Meta-analysis between genetic ancestry (square). Subjects with COPD were those with moderate-to-severe disease (FEV_1/FVC<0.7 and FEV_1%predicted <80%). Error bars in forest plot show 95% confidence intervals.

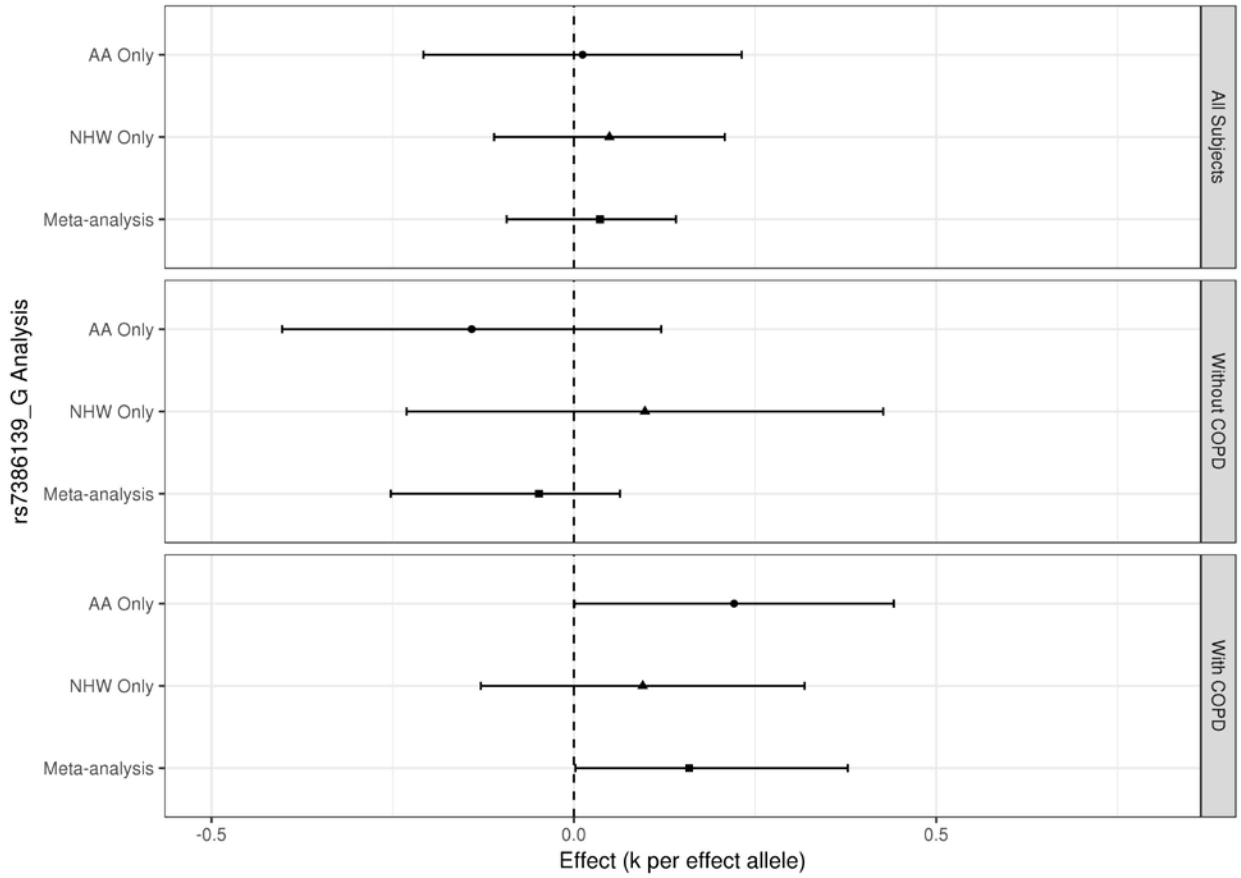

Figure S3. The effect on muscle oxygen consumption recovery rate constant (k) of G/A genetic variation on rs7386139 *DEPTOR* among all subjects in study, those without COPD and those with moderate-to-severe COPD stratified by genetic ancestry. AA (circle) = African American. NHW (triangle) = Non-Hispanic White. Meta-analysis between genetic ancestry (square). Subjects with COPD were those with moderate-to-severe disease ($FEV_1/FVC<0.7$ and FEV_1%predicted <80%). Error bars in forest plot show 95% confidence intervals

.

REFERENCES

1. **Adami A, Cao R, Porszasz J, Casaburi R, Rossiter HB**. Reproducibility of NIRS assessment of muscle oxidative capacity in smokers with and without COPD. *Respir Physiol Neurobiol* 235:18-26, 2017.
2. **Chang CC, Chow CC, Tellier LC, Vattikuti S, Purcell SM, Lee JJ.** Second-generation PLINK: rising to the challenge of larger and richer datasets. *Gigascience* 4:7, 2015.
3. **Chang X, Wang K.** wANNOVAR: annotating genetic variants for personal genomes via the web. *J Med Genet* 49:433-6, 2012.
4. **Ferrari M, Mottola L, Quaresima V.** Principles, techniques, and limitations of near infrared spectroscopy. *Can J Appl Physiol* 29:463-487, 2004.
5. **Kanda M, Minakata Y, Matsunaga K, Sugiura H, Hirano T, Koarai A, Akamatsu K, Ichikawa T, Ichinose M.** Validation of the triaxial accelerometer for the evaluation of physical activity in Japanese patients with COPD. *Intern Med* 51:369-375, 2012.
6. **Miller MR, Crapo R, Hankinson J, Brusasco V, Burgos F, Casaburi R, Coates A, Enright P, van der Grinten CP, Gustafsson P, Jensen R, Johnson DC, MacIntyre N, McKay R, Navajas D, Pedersen OF, Pellegrino R, Viegi G, Wanger J**. ATS/ERS Task Force. General considerations for lung function testing. *Eur Respir J* 26:153-161, 2005.
7. **Monteiro F, Camillo CA, Vitorasso R, Sant'Anna T, Hernandes NA, Probst VS, Pitta F.** Obesity and physical activity in the daily life of patients with COPD. *Lung* 190:403-410, 2012.
8. **Pitta F, Troosters T, Spruit MA, Decramer M, Gosselink R.** Activity monitoring for assessment of physical activities in daily life in patients with chronic obstructive pulmonary disease. *Arch Phys Med Rehabil* 86:1979-1985, 2005.
9. **Regan EA, Hokanson JE, Murphy JR, Make B, Lynch DA, Beaty TH, Curran-Everett D, Silverman EK, Crapo JD.** Genetic epidemiology of COPD (COPDGene) study design. *COPD: Journal of Chronic Obstructive Pulmonary Disease* 7:32-43, 2010.
10. **Ryan TE, Brophy P, Lin CT, Hickner RC, Neufer PD**. Assessment of in vivo skeletal muscle mitochondrial respiratory capacity in humans by near-infrared spectroscopy: a comparison with in situ measurements. *J Physiol* 592:3231-41, 2014.
11. **van Remoortel H, Raste Y, Louvaris Z, Giavedoni S, Burtin C, Langer D, Wilson F, Rabinovich R, Vogiatzis I, Hopkinson NS, Troosters T**. Validity of six activity monitors in chronic obstructive pulmonary disease: a comparison with indirect calorimetry. *PLoS ONE* 7:e39198, 2012.

12. **Wang K, Li M, Hakonarson H.** ANNOVAR: functional annotation of genetic variants from high-throughput sequencing data. *Nucleic Acids Res* 38:e164, 2010.
13. **Willer CJ, Li Y, Abecasis GR.** METAL: fast and efficient meta-analysis of genomewide association scans. *Bioinformatics* 26:2190-2191, 2010.
14. **Wüst RC, van der Laarse WJ, Rossiter HB.** On-off asymmetries in oxygen consumption kinetics of single Xenopus laevis skeletal muscle fibres suggest higher-order control. *J Physiol* 591:731-44, 2013.
15. **Yang H, Wang K.** Genomic variant annotation and prioritization with ANNOVAR and wANNOVAR. *Nat Protoc* 10:1556-1566, 2015.
